# The Epidemiology of Alzheimer's Disease Modifiable Risk Factors and Prevention

**DOI:** 10.14283/jpad.2021.15

**Published:** 2021-04-10

**Authors:** X.-X. Zhang, Y. Tian, Z.-T. Wang, Y.-H. Ma, Lan Tan, Jin-Tai Yu

**Affiliations:** 1 Department of Neurology, Qingdao Municipal Hospital, Qingdao University, No.5 Donghai Middle Road, Qingdao, China; 2 Department of Neurology and Institute of Neurology, Huashan Hospital, Shanghai Medical College, Fudan University, No. 12 Wulumuqi Road, Shanghai, China

**Keywords:** Alzheimer's disease, epidemiology, modifiable risk factors, prevention

## Abstract

Mild Alzheimer's disease is the leading cause of dementia, accounting for 50–70% of cases. Alzheimer's disease is an irreversible neurodegenerative disease, which affects daily life activities and social functioning. As life expectancy increases and demographic ageing occurs, the global prevalence of Alzheimer's disease is expected to continue to rise especially in developing countries, leading to a costly burden of disease. Alzheimer's disease is a complex and multifactorial disorder that is determined by the interaction of genetic susceptibility and environmental factors across the life course. Epidemiological studies have identified potential modifiable risk and protective factors for Alzheimer's disease prevention. Moreover, Alzheimer's disease is considered to start decades earlier before clinical symptoms occur, thus interventions targeting several risk factors in non-demented elderly people even middle-aged population might prevent or delay Alzheimer's disease onset. Here, we provide an overview of current epidemiological advances related to Alzheimer's disease modifiable risk factors, highlighting the concept of early prevention.

## Introduction

**A**lzheimer's disease (AD), as the most prevalent cause of dementia, is defined by deterioration in cognition, function and behavior, which typically begins in memory loss about recent events (1). The decisive pathological features in AD patients' brain tissues are raised levels of both amyloid-β (Aβ) composing of extracellular senile plaques and hyperphosphorylated tau (p-tau) aggregating intracellularly as neurofibrillary tangles (NFTs) (2). About 50 million people are living with dementia around the world, due to the aging population, the number of patients is predicted to triple by 2050, which increases the risk of disability, burden of illness and health care costs (3). Moreover, current treatment strategies only ameliorate symptoms and there is no effective cure for AD. However, AD has a long prodromal period during which early prevention appears to be particularly important to slow down the progression of AD. Therefore, epidemiological investigations are essential to identify risk and protective factors that strongly influence cognitive status. In fact, one-third of AD cases worldwide are attributable to underlying modifiable risk factors (4), which might modulate an individual's risk of developing AD. In this review, we classified these factors into several categories including psychosocial factors, pre-existing diseases, lifestyles and others, exploring the potential effects on cognition to provide better implications for AD prevention.

## Descriptive epidemiology of Alzheimer's disease

The number of dementia patients is projected to reach 152 million by mid-century worldwide, with the greatest increase expected in low-and middle-income countries (3). According to 2020 Alzheimer's disease facts and figures, the number of AD patients (≥ 65 years) might increase greatly from 5.8 million to 13.8 million by 2050 in America (5). The obviously increased AD prevalence was found in community-dwelling investigations of Japan and China over the last few decades (6, 7). Particularly, age-specific global prevalence in women was 1.17 times larger than in men and the age-standardized mortality rate of women was also higher than men, suggesting the longer lifespan was not the only determinant of the women dominance (8). In addition, death tolls with AD increased 146.2% from 2000 to 2018 and AD became the fifth-largest cause of death in American old people (5). Notably, caregivers would experience more mental stresses and negative emotional influences (5). Therefore, the social and family burden of caring for AD population will be huge and unsustainable.

Wellbeing is the aim of much of AD care. The AD patients would have perplexing problems and symptoms in many domains. And some epidemiological investigations have provided robust evidence that behavioral and environmental factors have key roles in disease pathogenesis and progression. Especially, preexisting disease is more common in AD patients than others of the same age, it is essential to keep physically healthy to protect the cognition. In addition, many risk factors could contribute to the development of AD and also be regarded as the symptoms of AD simultaneously, the reverse causality might account for this. Therefore, the accurate diagnosis is so important for those individuals suffering from cognitive dysfunction. Though AD is indicated by Aβ and tau biomarkers, some cognitively normal individuals having only these biomarkers never develop AD (9), it means that the pre-symptomatic diagnosis is more difficult to obtain. Future challenges would include discovering less-invasive and more-sensitive biomarkers or methods that can also be used for early screening and diagnosis purposes. Anyway, evidence-based prevention strategies, in line with the potential link between modifiable risk factors and late-onset AD, need to be explored in future studies.

## Putative modifiable risk factors and prevention for late-onset Alzheimer's disease

**Figure 1:**
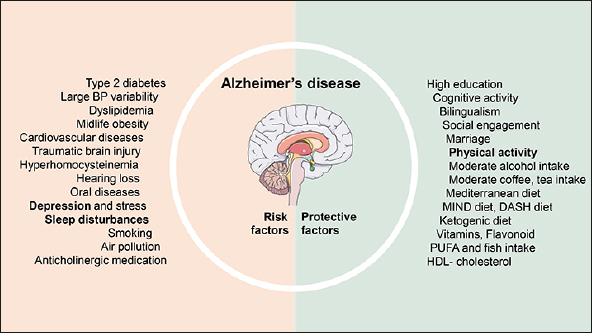
Potential modifiable risk factors for Alzheimer's disease Risk factors mainly included pre-existing diseases, unhealthy lifestyles and environmental exposures, while some factors concerning psychosocial conditions as well as healthy lifestyles might protect against AD. In addition, some factors appeared to be risk factors as well as symptoms of AD, possibly due to the reverse causality, these factors were highlighted in bold. Abbreviation: BP = blood pressure, DASH = Dietary Approach to Stop Hypertension, MIND = Mediterranean-DASH diet Intervention for Neurodegeneration Delay, PUFA = polyunsaturated fatty acid, HDL- cholesterol = high-density lipoprotein cholesterol.

**Figure 2:**
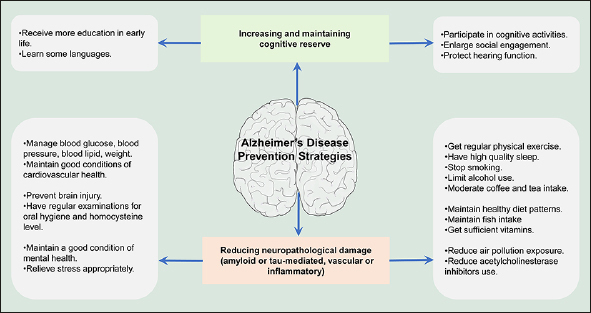
Implications for preventing Alzheimer's disease and slowing its progression It is imperative to increase the cognitive reserve mainly via enhancing education attainment and promoting social contact. Additionally, good conditions of body health and healthy lifestyles as well as reducing environmental exposures might be favorable to reduce the neuropathological damage for AD prevention.

Evidence from observational studies has accumulated during the past few years and shown several potentially modifiable risk factors (Figure 1), concerning AD prevention some potential feasible suggestions are provided (Figure 2).

### Psychosocial factors

Some prospective cohorts have studied whether these psychosocial factors might affect the cognition specifically (Supplemental Table 1).

### Educational attainment

Continuing adult education could benefit language processing and intellectual capacities (10), long-term education might also have favorable influences on total brain volume such as elevated cortical surface area and thickness in the prodromal stage of AD (11, 12). Mendelian randomization studies confirmed causal associations between education and reduced AD risk [odds ratio (OR) 0.64, 95% confidence intervals (CI) 0.56–0.74] as well as delayed AD onset [hazard ratio (HR) 0.76, 95% CI 0.67–0.85] (12), which could be mediated by intelligence in part (13). These causal relationships should be delineated profoundly in future studies.

#### Cognitive activity and bilingualism

Active engagements in cognitive activity were likely to have 46% reduction of AD risk in the Swedish study (14). Specifically, reduced decline in memory ability and cognitive speed among those playing more analog games was reported in the Lothian cohort study (15), which could be explained in part by intensive connectivity between the hippocampus and superior frontal cortex (16). Bilingualism, as a constituent of cognitive reserve, could enhance neural efficiency by increased functional connectivity in the frontoparietal control network for executive control and the default mode network for behavior control (17), certainly, bilinguals had stronger executive and visual-spatial functions than monolinguals (18). Specifically, compared with those only speaking Cantonese or Mandarin, lifelong bilinguals manifested the first symptoms of AD markedly later (19).

Higher educational attainment could delay the onset of AD by building cognitive reserve and brain volume (11). Similarly, cognitive activity and bilingualism were also beneficial to preserve the healthy cognitive functioning (14, 15, 18). Therefore, it is needed to increase access to education especially language and promote cognitive activities in the general population to protect against AD.

#### Social engagement

Frequent social contact with friends was related to a modestly decrease in dementia risk, owing to the creation of cognitive reserve at the early-stage via regular heathy social engagement (20). Particularly, community cultural activities might also confer benefits to the whole cognitive function (21). A healthy couple relationship might exert greater protective effects on cognition, whereas the widowed, especially those APOE ε4 carriers, had higher risk of AD (OR 7.67, 95% CI 1.6–40.0) than those married people (22). Indeed, among cognitively intact older people, widowed individuals were at higher likelihood of suffering serious Aβ-related cognitive deterioration (23). More social engagements could exercise memory and language which might further increase cognitive reserve (20). Of note, the widowed or the singled seemed to have less communication with others, it is encouraging to establish healthy social relationships and engage more regular social activities.

#### Depression and stress

Depression was a significant risk for AD (OR 1.65, 95% CI 1.42–1.92) (24). The finding of Australian study further proposed that the dementia odds ratios of mild, moderate to severe depressive symptoms were 1.2 (95% CI 1.0–1.4), 1.7 (95% CI 1.4–2.2) and 2.1 (95% CI 1.4–3.2). In addition, treatment with the citalopram for more than 4 years was strongly linked to a 3-year delay in progression from mild cognitive impairment (MCI) to AD (25). Notably, citalopram could reduce cerebrospinal fluid (CSF) amyloid plaque in experimental transgenic AD mice and healthy volunteers (26), conferring great significance to clinical practice. Besides, greater microglial activation, a symbol of neurodegenerative inflammation, existed in depressive patients not receiving medications (27), thus, reasonable treatment of depression might mean significant to prevent from neurodegeneration. However, depression and AD might have common causes such as inflammation and neurodegeneration, or similar symptoms in the prodromal stage of AD (28), thus depressive symptoms appeared to be a prodromal marker of AD rather than a causal risk factor. Further, the causal association of depression with AD was not supported in a mendelian randomization study (12), therefore, this association should be explored more in detail in future studies.

Higher risk of AD incidence (HR 1.36, 95% CI 1.12–1.67) was found among those individuals with stress-related disorders (29). Chronic work-related stress was also an emerging risk factor for AD (30). Notably, oxidative stress and neuroinflammation could be induced by stress, which could further promote the production of amyloid plaques (31). Regulating stress and adjusting emotions would ameliorate detrimental psychological effects on cognition.

### Pre-existing diseases

We concluded detailed discoveries of prospective studies concerning the relationships between all these pre-existing diseases and AD development (Supplemental Table 2).

#### Diabetes, hypertension and dyslipidemia

Evidence supporting the higher risk of affecting with AD among the diabetics [relative risks (RR) 1.43, 95% CI 1.25–1.62] was based on 24 longitudinal studies (32). Specifically, a steeper fall in perceptual speed and verbal abilities among the diabetics was also found in the Swedish study (33). Interestingly, the interaction of Aβ with islet amyloid polypeptide exacerbating AD pathogenesis might explain the molecular association of diabetes with AD (34). The diabetics without proper treatment had higher CSF p-tau level than those using antidiabetic drugs or euglycemic adults, suggesting tau pathology might be ameliorated with the antidiabetic drugs (35). Moreover, metformin use and treatment with anti-diabetic drugs might slow down the development of cognitive decline and reduce the risk of dementia (36, 37).

A remarkably increase in AD risk (HR 1.73, 95% CI 1.02–2.94) among those with midlife and late-life high systolic pressure has also been reported in the Framingham Offspring Study (38). However, decreased systolic pressure from mid-to late life also contributed to higher risk of AD (HR 2.12, 95% CI 1.12–4.00) (38). Individuals with large blood pressure variability exhibited a larger than 2-fold risk of AD (39), indicating excessive blood pressure variation might partially be responsible for AD deterioration. Fortunately, the AD risk was decreased (RR 0.78, 95% CI 0.66–0.91) because of the treatment of antihypertensives, longer use of these drugs might have significantly protective effects on cognition (40).

Blood lipids such as total cholesterol (TC), triglycerides (TG), low-density lipoprotein cholesterol (LDL-C) and high-density lipoprotein cholesterol (HDL-C) might play important parts in the process of AD. The Three-City Study reported a significant increase in AD risk among those with hypercholesterolemia, particularly, this association would be weakened after adjusting for APOE ε4 (41). In addition, the cholesterol was divided into two subtypes including HDL-C and non-HDL-C in the Adult Changes in Thought (ACT) Study, the finding revealed a U-shaped association of non-HDL-C level with risk of AD among 60–69 aged individuals, compared with 160 mg/dl non-HDL-C, the HR was 1.29 (95% CI 1.04–1.61) at 120 mg/dl and 1.16 (95% CI 1.01–1.33) at 210 mg/dl (42). Notably, hypercholesterolemia could compromise the integrity of the blood-brain barrier, increase Aβ deposition, as well as cause neuroinflammation, all of which could exacerbate the development of AD (43). In addition, increased LDL-C levels were associated with higher AD risk (41), while HDL-C was likely to be a protective factor against AD (44). There existed no significant association between AD risk and TG level (41). Encouragingly, a meta-analysis indicated that uses of stain drugs were likely to lower the risk of AD (RR 0.86, 95% CI 0.80–0.92) among those with hyperlipidemia in prospective studies, but these beneficial effects were not stronger in randomized controlled trials (40).

Young adults had better stay away from diabetes, hypertension, hypercholesterolemia via healthier lifestyles. Patients could manage these diseases via regular uses of pharmaceuticals. Taking regular antidiabetics was associated with less tau dysfunction delaying the progression of AD (35). The antihypertensive drugs and stain drugs might be favorable to lower the risk of AD among those patients (40, 45). More studies are required to investigate whether particular medications would effectively lower the risk of developing AD.

#### Obesity

Consistent evidences have proposed a higher risk of developing AD among middle-aged patients with raised body mass index (BMI) whereas overweight or obesity in late-life might have protective effects on cognition (46, 47). Particularly the HR for AD was 0.89 (95% CI 0.81–0.98) among those with high late-life BMI, in contrast, there was 20% increased risk for AD (95% CI 1.09–1.33) among those with greater loss of BMI from midlife to late-life (46). Consistently, the obese elderly tended to have less Aβ load and larger hippocampus volume (47). The underlying mechanism might be partially attributed to the increased leptin produced mainly by adipocytes, leptin could robustly facilitate the neurogenesis of hippocampus in AD mouse models (48). Of note, we should emphasize the role of age in evaluating the association of obesity with AD. Young adults should maintain or lose weight under the healthy range (18.5–24.9 kg/m^2^) while older individuals should avoid greater weight loss otherwise attention should be focused on this situation.

#### Cardiovascular diseases

The HR of AD following atrial fibrillation was 1.31 (95% CI 1.20–1.43) in the Korean study (49). In addition, mild reductions in cardiac index were also related to a marked increase in AD risk (HR 2.87, 95% CI 1.21–6.80) over a long period of 7.7 years (50). Cerebral hypoperfusion, caused by lower cardiac output in atrial fibrillation patients, could explain the deteriorations of cortical atrophy and neuropathological features (51). Encouragingly, individuals with atrial fibrillation undergoing oral anticoagulants were at a lower risk of developing AD (HR 0.61, 95% CI 0.54–0.68) (49). Interestingly, compared with medical therapy, the risk of AD was decreased among atrial fibrillation patients treated with catheter ablation (HR 0.77, 95% CI 0.61–0.97) (52).

About 59% elevated risk of AD among stroke patients was well documented in a meta-analysis incorporating six studies (53). Consequently, effective strategies to reduce the extent of brain injury after stroke may help delay or prevent the progression of AD. A markedly increase in AD risk among individuals with multiple cerebral microbleeds has been reported in the Rotterdam Study (HR 2.10, 95% CI 1.21–3.64) (54). Meanwhile, severe cerebral atherosclerosis (OR 1.33, 95% CI 1.11–1.58) and arteriolosclerosis (OR 1.20, 95% CI 1.04–1.40) were also regarded as strong risk factors for AD (55). Neurovascular unit disorder, due to cerebrovascular diseases, could cause decreased cerebral blood flow, induce blood-brain barrier disruptions as well as selective brain atrophies, all of which could result in direct damage to neurons and trigger Aβ accumulation indirectly (56). Thus, better understanding of these relationships may acquire cognitive benefits from appropriate treatment.

Early screening and intervention of vascular risks as well as maintaining good cardiovascular conditions should become the top priority for AD prevention. Pharmaceuticals uses like oral anticoagulants and catheter ablation would significantly lower the risk of AD among atrial fibrillation patients (49, 52).

#### Traumatic brain injury

Participants exposed to traumatic brain injury (TBI) had increased risk of developing AD in the Danish study (HR 1.16, 95% CI 1.12–1.22) and in a Swedish study (OR 1.58, 95% CI 1.49–1.69), which could be strengthened by some detailed discoveries of TBI such as severe and multiple TBIs, the first few months since the trauma occurrence, younger individuals with the injury as well as TBI involving the skull or spine (57, 58). Particularly, athletes experiencing many years of head injury were more susceptible to die from AD according to their death certificates (59). Furthermore, among the American military veterans, women having TBI were at higher risk of developing dementia (60). Encouragingly, stain medication especially the rosuvastatin, owing to the potential neuroprotective benefits, might reduce the dementia risk among those individuals having a concussion (45). Thus, more studies are required to focus on the risk of TBI individuals to have AD and formulate therapeutic strategies to mitigate the risk and impact of AD. Considering the deleterious effects of TBI on cognition, the public had better take measures to protect the head properly from injuries when engaging in dangerous activities or work.

#### Hyperhomocysteinemia

A meta-analysis incorporating 5 prospective studies found a linear dose-response relationship between blood homocysteine (Hcy) levels and risk of AD (HR 1.15, 95% CI 1.04–1.26, per 5 *µ*mol/L increment) (61). However, in some findings of longitudinal studies such dose-dependent relationship only existed in the range of high serum Hcy concentrations (about ≥10 *µ*mol/l) (62). These inconsistent exposure-response associations should be further assessed in large-scale prospective studies. In addition, the elevation of Aβ deposition and tau hyperphosphorylation could be modulated by high Hcy levels via γ-secretase pathway and cdk5 kinase in mouse models (63). Additionally, hyperhomocysteinemia could be alleviated via folic acid supplementation (64), improving total homocysteine metabolism may also represent a viable strategy for AD prevention.

#### Hearing loss and oral diseases

And in a case-control study the OR was 1.39 for AD (95% CI 1.05–1.84) following hearing loss (65). Owing to the awful listening conditions, individuals might have difficulty in understanding speech and experience communication disorders even social isolation, contributing to reduced cognitive stimulation from the acoustic environment (66), which could aggravate cognitive impairment mediated by accelerated brain atrophy. Indeed, individuals with midlife hearing impairment tended to have prominent temporal lobe volume loss (67). Encouragingly, hearing aids and cochlear implants could mitigate some worse listening status, slowing down the rate of cognitive decline (68), early screening and correction of hearing loss might hold significant influence on AD prevention.

Oral diseases particularly tooth loss and chronic periodontitis were great concerns for cognitive dysfunction, probably mediated by local and systemic inflammatory responses (69), tooth loss was a strong risk factor for AD in the Hisayama Study (69). Specifically, individuals with chronic periodontitis were more susceptible to AD (HR 1.707, 95% CI 1.152–2.528) (70), periodontitis might induce peripheral inflammation through byperiodontal pathological bacteria directly or proinflammatory cytokines indirectly (71). Much attention should be attached to oral care especially in developing counties to prevent AD.

### Lifestyles

We identified plenty of prospective studies regarding lifestyles related to the risk of cognitive decline and AD (Supplemental Table 3, Supplemental Table 4).

#### Physical activity

Higher participation in daily physical activity was related to about half decreased risk of AD in the Hisayama Study (72). In addition, cognitive benefits of regular resistance exercise and choreographic intervention could delay the neurodegenerative process especially in those domains related to the conversion to dementia (73, 74). Besides, aerobic exercise was also beneficial to some cognitive domains including executive function and oral fluency (75), possibly due to the protective effects on hippocampus volume and neuronal health (76). After one-year aerobic exercise training, improvements of cardiorespiratory function particularly cerebral perfusion and memory ability were found in a prospective study (77). The neurotrophic effects of active exercise are needed to be further investigated, especially its type, intensity, duration and timing might have greater implications on AD prevention. However, in the Whitehall II study physical activities appeared to have no neuroprotective effects on cognitive functioning, individuals in the preclinical stage of AD tended to have lower physical activity levels than the healthy elderly (78), in other words, reverse causality might account for the relationship between active exercise and reduced risk of AD. It still remains controversial whether reverse causality could explain the favorable effects of physical activity on cognition.

#### Sleep disturbances

Sleep disturbances (insomnia and sleep disordered breathing) were risk factors for AD, and those with sleep disorders were more likely to experience more accumulation of neurotoxic substances as a result of the decreased metabolite clearance ability (79). There was a 66% (95% CI 1.03–2.68) increased AD risk in participants having severe obstructive sleep apnea (≥30 vs.<5 apnea-hypopnea events/hour) (80). Additionally, individuals with longer sleep length (> 9 hours) showed a greater than 2-fold risk of AD (81), the RR of AD following habitual shorter sleep duration was 1.25 (95% CI 0.88–1.76) (80). Moreover, both short duration (≤6 hours) and long duration (≥8 hours) all had detrimental effects on cognitive function, it further offered a V-shaped association of daily sleep duration with cognitive decline and subsequent risk for dementia (82). Notably, greater amyloid deposition was more common among those who had insufficient or excessive nocturnal sleep time (83). And there existed a causal relationship between sleep length and elevated cortical thickness in a mendelian randomization study (12). There may also exist a bidirectional relationship between sleep dysregulation and AD pathology, sleep disorders could stimulate the accumulation of Aβ and tau, meanwhile the enhanced aggregation of Aβ and tau may exacerbate the progression of sleep disturbances (84). More long-term longitudinal studies are needed to further explore the potential role of sleep dysregulation as a biomarker of AD and the potential bidirectional relationship. High quality sleep is extremely important in maintaining cognition, when having sleep problems patients should consult a doctor and receive therapy in time.

#### Smoking

Smoking increased the risk of AD (RR 1.40, 95% CI 1.13–1.73), which was also prominent among non-APOE ε4 carriers in a meta-analysis including 37 longitudinal studies (85). Of note, smoking initiation was associated with lower cortical thickness (12). Smoking-related cerebral oxidative stress might facilitate the production of amyloid or tau pathology (86). In contrast, never-smokers had 18% risk reduction of AD than continual smokers (87), indicating that early smoking cessation would confer greater benefits on cognition.

#### Alcohol consumption

The effects of alcohol intake on cognitive function may remain controversial in many epidemiological findings. The Nord-Trøndelag Health study supported a 47% elevated AD risk (95% CI 1.00–2.16) among frequent alcohol drinkers (≥5 times/two weeks) vs. infrequently drinkers (1–4 times) (88). This relationship was thought to be J-shaped, suitable alcohol intake (<12.0 g/day) could protect against dementia (89, 90), similar with the relationship between coffee intake and AD. It is not advisable to take excessive drinking in daily life, as for the heavy alcohol drinkers, extracellular cold-inducible RNA-binding protein (eCIRP) might mediate tau phosphorylation, leading to the progression of alcohol-induced AD (91). After matured hop bitter acid supplementation from beer, improvement of cognitive status was confirmed in a randomized trial (92). Particularly, alcohol from wine appeared to be stronger inversely related to the risk of dementia (89, 90), polyphenolic and antioxidant contents in wine showed greater protective effects against neurodegeneration (93). More primary studies should be warranted to clarify the underlying mechanism explaining the AD risk related to alcohol intake.

The only established causal relationship between earlier AD onset and alcohol consumption was found in a mendelian randomization study (94), which indicated that moderate alcohol intake might be harmful to the brain health but not beneficial. This study also stressed that potential confounding factors should be seriously taken into consideration, especially the survival bias (94), therefore future studies should delineate this relationship thoroughly and precisely.

#### Coffee and tea

A J-shaped association of coffee intake with AD was proposed, low (1–2 cups/day) but not high (>3 cups/day) coffee intake was related to a 18% reduction of AD risk (RR 0.82, 95% CI 0.71–0.94, vs. <1 cup/day) (95), which was more pronounced among women (96). However, this gender characteristic was different from tea intake. The protective effects of green tea on cognition were more prominent in men (97, 98). Some neuroprotective components may exist in the coffee or green tea drinking like caffeine and L-theanine (99), anti-amyloid effects of green tea might protect against AD mainly including inhibition of Aβ aggregation and reduction of Aβ-induced oxidative stress (100). Moderate coffee consumption and green tea drinking should be encouraged to the public. There was reduced level of CSF total-tau protein among frequent green tea consumers, probably owing to improved abnormal tau metabolism (98). However, the black tea and oolong tea did not show cognitive benefits in the overall elderly Han study population (97).

#### Diets

Three dietary patterns including the Mediterranean diet (HR 0.46, 95% CI 0.26–0.79), the DASH (Dietary Approaches to Stop Hypertension) diet (HR 0.61, 95% CI 0.38–0.97) and the MIND (Mediterranean-DASH Intervention for Neurodegenerative Delay) diet (HR 0.47, 95% CI 0.26–0.76), were all inversely associated with the risk of AD (101), the protective effects of these dietary patterns on cognition might be attributed to anti-oxidant, anti-inflammatory, and anti-diabetic effects and enough mono-/poly-unsaturated fats (102). More interestingly, the Three-city study proposed the concept of novel diet pattern, a more diverse diet including vegetables, fresh fruits and seafood, might be particularly beneficial to the cognitive function (103). Particularly, ketogenic diets could reduce the AD risk via altering gut mycobiome (104), while following higher glycemic load intake the HR increased by 27% for AD (105). Additionally, a meta-analysis incorporating 21 cohort studies proposed that higher intake of fish (RR 0.93, 95% CI 0.90–0.95) and marine-derived dietary docosahexaenoic acid (RR 0.63, 95% CI 0.51–0.76) could protect against the risk of developing AD (106). Besides, habitual intake of seafood was associated with lower burden of AD brain pathology among APOE ε4 carriers, which was not affected by higher brain levels of mercury (107).

Severe 25-Hydroxyvitamin D [25(OH)D] deficiency (<25 nmol/L) was related to elevated risk of AD (HR 2.22, 95% CI 1.02–4.83), as was 25(OH)D inadequacy (25–50 nmol/L) (HR 1.69, 95% CI 1.06–2.69), using the level of more than 50 nmol/L as the refence category (108). Persons in early adulthood taking more B vitamins including niacin, folate, vitamin B-6, and vitamin B-12 were likely to acquire better cognitive performance in late life (109). In addition, the potential cognitive benefits of vitamin C were more remarkable among women APOE 4-carriers while higher blood vitamin E level might hold such benefits among APOE 4-negative men (110). Higher flavonoids intake from daily food might reduce the risk of AD incidence by 38% (95% CI 0.39–0.98), which was independent of other lifestyle factors and cardiovascular related diseases (111). Therefore, further studies are required to delineate the potential biologic explanations, and more mendelian randomization studies are needed to elucidate whether these associations of micronutrients intake with the cognition are causal. It is necessary to take certain vitamins and other micronutrients from daily diets properly.

### Others

#### Medications

Current or former nonsteroidal anti-inflammatory drugs (NSAIDs) exposure was associated with a 19% reduction in AD risk (95% CI 0.70–0.94) (112). However, in a randomized placebo-controlled trial, no robust evidence supported that aspirin could effectively lower the risk of AD (113), aligned with the meta-analysis conducted by Veronese et al (114). In this meta-analysis, low-dose aspirin did not seem to improve the cognitive function (114). Therefore, it still remains controversial whether NSAIDs would protect against AD.

The adverse impact of anticholinergic medications on cognition was underscored. Over a long period of 4 years, excess use of anticholinergics was considered as a risk for AD (HR 1.63, 95% CI 1.24–2.14) (115). Minimizing anticholinergic use over time might be important to preserve the cognition.

#### Pollutions

We identified several longitudinal cohorts studying some environment factors associated with the risk of cognitive decline and AD (Supplemental Table 3). Misfolding and abnormal aggregation of p-tau and Aβ were found in the brainstem of children and young adults exposed to Mexico City's air pollution (116). In a study from Northern Sweden, there was a 38% elevated risk for AD among those exposed to residential traffic-related air pollution (95% CI 0.87–2.19) when comparing the highest with the lowest quartile of NOx (≥26 vs. ≤9 µg/m3) (117). Indeed, Particulate Matter2.5 (PM2.5) relevant with gray matter atrophy indicated higher risk of AD (HR 1.24, 95% CI 1.14–1.34) in older women (118, 119). The adverse effects of air pollutants on cognition might be amplified by cardiovascular diseases including heart failure and ischemic heart disease (120). Crucially, long-term air pollution might accelerate the progression of neurodegeneration via vascular disease, Aβ deposition and neuroinflammation (116, 120). Therefore, reducing the exposures of air pollution seems particularly important for AD prevention. More studies are required to investigate the effects of environmental exposures on AD and the potential mechanisms underlying these relationships.

## Conclusions

Many longitudinal studies have identified various risk and protective factors for AD, including some that could be targeted to reduce risk of AD or delay the onset of AD, suitable preventions might help slow down the progress of AD. More policies targeting education popularization and social or cognitive activities promotion should be put forward among the public. Managing the preexisting disease reasonably and maintaining daily healthy lifestyles would protect against AD. Additionally, environment protection especially targeting air pollutants would be of great importance to AD prevention. If possible, more studies should focus on individuals at high risk of AD or in the prodromal stage of AD, among whom daily preventions and neuroprotective interventions are likely to exert greater favorable effects.

## Electronic supplementary material

Supplementary material, approximately 190 KB.

*Conflicting interests:* None.
