# Supplementary material for: The Epidemiology of Alzheimer's Disease Modifiable Risk Factors and Prevention
Source: J Prev Alzheimers Dis. 2021 Apr 10;8(3):313–21. doi: 10.14283/jpad.2021.15 (PMC12280729; doi:10.14283/jpad.2021.15)
Supplement: Supplementary file 1 — Supplementary material, approximately 190 KB. [file mmc1.docx]

**Supplemental Table 1.** **Prospective studies of psychosocial factors in associations with cognitive decline and Alzheimer’s disease**

| **Author** | **Publication year** | **Studies** | **Follow-up(years)** | **No. of participants** | **Age at baseline (years)** | **Female (%)** | **Risk factor measurements** | **Risk factor** | **Outcomes** |
| --- | --- | --- | --- | --- | --- | --- | --- | --- | --- |
| Gottesman, R. F.(1) | 2017 | The Atherosclerosis Risk in Communities Cohort | 25 | 15,744 | 44-66 | 55 | In-person visits, hospitalization surveillance,  telephone calls | Lower educational attainment | With increased risk of dementia |
| Wilson, R. S.(2) | 2012 | The Rush Memory and Aging Project | 5 | 1,076 | Mean 80 | 74 | Annual evaluation using a 5-point scale | More frequent mental stimulation | With better cognitive functioning |
| Najar, J.(3) | 2019 | The Gothenburg H70 Birth Cohort Studies | 44 | 800 | Mean 47 | 100 | Semi-structured psychiatric interviews | Cognitive  activity | With a reduced risk of AD |
| Marioni, R. E.(4) | 2015 | The Paquid cohort | 20 | 2,854 | Mean 77 | 59 | A scale for late-life social, intellectual engagement | Social engagement | With a decreased risk of dementia |
| Zhou, Z.(5) | 2018 | The Chinese Longitudinal Healthy Longevity Survey | 9 | 7,511 | ≥65 | 54 | Questions | Social engagement | With a lower risk of dementia |
| Amieva, H.(6) | 2010 | The Paquid cohort | 15 | 2,089 | ≥65 | 60 | Questionnaire | Social network | With a reduced risk of AD |
| Sommerlad, A.(7) | 2019 | The Whitehall II cohort study | 28 | 10,228 | 35-55 | 33 | A self-report questionnaire | Social contact | Conferring higher cognitive reserve |
| Fancourt, D.(8) | 2020 | The English Longitudinal Study of Ageing | 12 | 9,550 | >50 | 45 | Questions | Community engagement | With a lower risk of developing dementia |
| Biddle, K. D.(9) | 2020 | The Harvard Aging Brain Study | 4 | 257 | Mean 74 | 60 | Self-report | Marital status (the widowed) | With accelerated β-amyloid-related cognitive decline |
| Saito, T.(10) | 2018 | The Aichi Gerontological Evaluation Study prospective cohort | 9 | 13,984 | ≥65 | 51 | Questions | Social relationship | With decreased risk of dementia |
| Geda, Y. E.(11) | 2014 | The Mayo Clinic Study of Aging | Median 5 | 1,587 | Median 80 | 50 | NPI-Q | Depression | With an increased risk of MCI |
| Holmquist, S.(12) | 2020 | The Swedish National Patient Register (Matched cohort) | 11 | 238,772 | >50 | 61 | ICD | Depression | With increased risk of dementia |
| Holmquist, S.(12) | 2020 | The Swedish National Patient Register (The sibling cohort) | >20 | 50,644 | >50 | 58 | ICD | Depression | With increased risk of dementia |
| Almeida, O. P.(13) | 2016 | The Health in Men Study | 5 | 4,568 | 70-89 | 0 | The GDS-15 | Depressive symptoms | With increased risk rate of cognitive impairment |
| Almeida, O. P.(14) | 2017 | The Health in Men Study | 14 | 4,922 | 71-89 | 0 | Questions and the Geriatric Depression Scale | Depression | With increased risk of developing dementia |
| Steenland, K.(15) | 2012 | The large National Alzheimer’s Coordinating Center database | Mean 3 | 8,855 | 73 | 61 | The GDS-15 or the NPI-Q | Depression | With an increased risk of MCI |
| Song, H.(16) | 2020 | The Swedish National Patient Register (Matched cohort) | About 5 | 657,083 | <50 | 61 | ICD-9 | Stress-related disorders | With increased risk of AD |
| Song, H.(16) | 2020 | The Swedish National Patient Register (The sibling cohort) | About 5 | 123,321 | <50 | 54 | ICD-9 | Stress-related disorders | With increased risk of AD |
| Sindi, S.(17) | 2017 | The CAIDE study | 30 | 2,000 | Mean 50 | 63 | Questions | Midlife work-related stress | With an increased risk of AD |
| Wang, H. X.(18) | 2012 | The Kungsholmen Project | 6 | 913 | ≥75 | 0 | Questions | Psychosocial stress at work | With an increased risk of AD |
| Gradus, J. L.(19) | 2018 | The Danish National Patient Registry | 17 | 279,188 | ≥40 | NA | The national Danish Psychiatric Central Research Registry | Stress Disorders | With higher risk of dementia |
| Flatt, J. D.(20) | 2018 | The Kaiser Permanente Northern California health system | Mean 8 | 499,844 | >60 | 55 | ICD-9 | Post-traumatic stress disorder | With increased risk of dementia |
| Wang, T. Y.(21) | 2016 | The Taiwan National Health Insurance Research Database | About 7 | 8,750 | >55 | 77 | ICD-9 | Post-traumatic stress disorder | With higher risk of developing dementia |

The CAIDE study = The Cardiovascular Risk Factors, Aging and Dementia study; AD = Alzheimer’s disease; MCI = mild cognitive impairment; NPI-Q=Neuropsychiatric Inventory Questionnaire; ICD-9 = International Classification of Diseases-9th Revision; GDS-15 = Geriatric Depression Scale 15

**Supplemental Table 2.** **Prospective studies of pre-existing diseases in associations with cognitive decline and Alzheimer’s disease**

| **Author** | **Publication year** | **Studies** | **Follow-up(years)** | **No. of participants** | **Age at baseline (years)** | **Female (%)** | **Risk factor measurements** | **Risk factor** | **Outcomes** |
| --- | --- | --- | --- | --- | --- | --- | --- | --- | --- |
| Marseglia, A.(22) | 2019 | The Swedish National Study on Aging and Care-Kungsholmen | 9 | 2,746 | ≥60 | 63 | Using High Performance Liquid Chromatography to measure HbA1c levels and self-report | Diabetes and prediabetes | With faster cognitive decline |
| Zheng, F.(23) | 2018 | The English Longitudinal Study of Ageing | Mean 8 | 5,189 | Mean 66 | 55 | Using a Tosoh G7 analyser to measure HbA1c levels | Diabetes | With faster cognitive decline |
| Marseglia, A.(24) | 2018 | The Swedish Adoption/Twin Study of Aging | 23 | 793 | ≥50 | 59 | Using an enzymatic method to test blood glucose level and self-report | Prediabetes and diabetes | With faster cognitive decline |
| McGrath, E. R.(25) | 2017 | The Framingham Offspring Study | Mean 8 | 1,440 | Mean 69 | 47 | Recorded in a standardized fashion | Systolic hypertension, decreased SBP | With an increased risk of AD |
| Rajan, K. B.(26) | 2018 | The Chicago Health and Aging Project | 18 | 2,137 | ≥65 | 63 | Using mercury sphygmomanometers and using digital oscillometric devices | Late life BP | With a U-shaped association of AD |
| Oishi, E.(27) | 2017 | The Hisayama study | 5 | 1,674 | ≥60 | 56 | Using a validated digital electronic device based on the cuff oscillometric method | BPV | With an increased risk of AD |
| Yoo, J. E.(28) | 2020 | The Korean National Health Insurance Service | 6 | 23,503,802 | Mean 55.5 | 48 | Measured by a trained clinician and using the average of the two brachial BP | BPV | With an increased risk of AD |
| Schilling, S.(29) | 2017 | The 3C Study | 13 | 7,470 | Mean 74 | 61 | Using enzymatic methods | Hypercholesterolemia, LDL-C | With an increased risk of AD |
| Marcum, Z. A.(30) | 2018 | The Adult Changes in Thought study | About 5 | 4,368 | ≥50 | 59 | Using all recorded measures in the laboratory database | non-HDL-C | With a U-shaped association of AD |
| Svensson, T.(31) | 2019 | The Japan Public Health Centre-based prospective Study | 19 | 1,299 | About 54 | NA | Using enzymatic methods | HDL-C | With a decreased risk of MCI |
| Tolppanen, A. M.(32) | 2014 | The CAIDE study | 26 | 1,304 | Mean 50, 71and 79 | 61 | BMI measurements | Late-life high BMI | With a decreased risk of AD |
| LeBlanc, E. S.(33) | 2017 | The Study of Osteoporotic Fractures | 20 | 1,289 | Mean 68 and 88 | 100 | BMI measurements | Late life weight loss | With an increased risk of MCI |
| Alhurani, R. E.(34) | 2016 | The Mayo Clinic Study of Aging | Mean 4 | 1,895 | ≥70 | 49 | BMI measurements | Late life weight loss | With an increased risk of MCI |
| Rusanen, M.(35) | 2014 | The CAIDE study | 26 | 1,510 | Mean 50 | 62 | Questionnaire | Atrial fibrillation | With an increased risk of AD |
| Dublin, S.(36) | 2011 | The ACT study | Mean 7 | 3,045 | Median 74 | 60 | ICD-9 | Atrial Fibrillation | With an increased risk of AD |
| Kim, D.(37) | 2019 | The Korea National Health Insurance Service Senior cohort | About 8 | 262,611 | ≥60 | 56 | ICD-10 | Atrial Fibrillation | With an increased risk of AD |
| Jefferson, A. L.(38) | 2015 | The Framingham Heart Study | Median 8 | 1,039 | Mean 69 | 53 | Brain MRI | Lower cardiac index | With an increased risk of AD |
| Akoudad, S.(39) | 2016 | The Rotterdam Study | Mean 6 | 3,257 | Mean 60 | 55 | Brain MRI | Cerebral microbleeds | With an increased risk of AD |
| Romero, J. R.(40) | 2017 | The Framingham Heart Study | Mean 7 | 1,296 | Mean 72 | 54 | Brain MRI | Cerebral microbleeds | With an increased risk of AD |
| Fann, J. R.(41) | 2018 | The Danish Civil Registration System | 14 | 2,794,852 | >50 | 52 | Using the Danish National Patient Register | Traumatic brain injury | With an increased risk of AD |
| Yaffe, K.(42) | 2019 | The Veterans Health Administration | Mean 4 | 109,140 | ≥55 | 100 | ICD-9 | Traumatic brain injury | With an increased risk of dementia |
| Chen, S.(43) | 2020 | The Hisayama Study, | 10 | 1,588 | 60-99 | 57 | Using the high- performance liquid chromatography method | High serum Hcy | With an increased risk of AD |
| Hooshmand, B.(44) | 2019 | The Swedish National Study on Aging and Care-Kungsholmen | 6 | 2,570 | Mean 73 | 57 | Using tandem mass spectrometry | High serum Hcy | With an increased risk of AD |
| Hooshmand, B.(45) | 2010 | The CAIDE study | 7 | 271 | 65-79 | 62 | Chemiluminescent microparticle immunoassay | High serum Hcy | With an increased risk of AD |
| Osler, M.(46) | 2019 | The Danish National Patient Registry | 39 | 942,567 | ≥65 | 0 | ICD-8, ICD-10 | Hearing loss | With an increased rate of dementia |
| Liu, C. M.(47) | 2019 | The National Health Insurance Research Database | 12 | 16,270 | 45-64 | 43 | ICD-9 | Hearing loss | With higher risk of dementia |
| Saito, S.(48) | 2018 | The Ohasama study | 4 | 140 | ≥65 | 69 | Counting the number of remaining teeth | Tooth loss | With an increased risk of MCI |
| Okamoto, N.(49) | 2015 | The Fujiwara-kyo Study | 5 | 2,335 | >65 | 48 | Using the blinded single observer method | Tooth loss | With an increased risk of MCI |
| Takeuchi, K.(50) | 2017 | The Hisayama Study | 5 | 1,566 | ≥60 | 56 | Following the Third National Health and Nutrition Examination Survey | Tooth loss | With an increased risk of AD |

The CAIDE study = The Cardiovascular Risk Factors, Aging and Dementia study; AD = Alzheimer’s disease; SBP = systolic blood pressure; BP = blood pressure; BPV = blood pressure variability; LDL-C = low-density lipoprotein cholesterol; HDL-C= high-density lipoprotein cholesterol; MCI = Mild cognitive impairment; BMI = body mass index; Hcy = homocysteine; ICD-9 = International Classification of Diseases-9th Revision; MRI = magnetic resonance imaging

**Supplemental Table 3.** **Prospective studies of environmental factors, lifestyle factors in associations with cognitive decline and Alzheimer’s disease.**

| **Author** | **Publication year** | **Studies** | **Follow-up(years)** | **No. of participants** | **Age at baseline (years)** | **Female (%)** | **Risk factor measurements** | **Risk factor** | **Outcomes** |
| --- | --- | --- | --- | --- | --- | --- | --- | --- | --- |
| Oudin, A.(51) | 2016 | The Betula study | ≥15 | 1,806 | ≥55 | 57 | Using a land-use regression model with a spatial resolution of 50 m × 50 m | Traffic-related air pollution | With higher risk of risk of AD |
| Chen, H.(52) | 2017 | The Ontario Population Health and Environment Cohort | ≥10 | 2,066,639 | Mean 67 | 53 | Using satellite observations, land-use regression model, and an optimal interpolation method | Ambient air pollution | With higher incidence of dementia. |
| Grande, G.(53) | 2020 | The Swedish National Study on Aging and Care in Kungsholmen | Mean 6 | 2,927 | Mean 74 | 63 | Using dispersion models for outdoor levels at residential addresses | Air pollution | With a higher risk of dementia |
| Oudin, A.(54) | 2018 | The longitudinal Betula study | ≥15 | 1,806 | ≥55 | 57 | Using a land-use regression model with a spatial resolution of 50 m × 50 m | Air pollution | With a higher risk of dementia |
| Kishimoto, H.(55) | 2016 | The Hisayama Study | 17 | 803 | ≥65 | 61 | Questionnaire | Leisure-time physical activity | With lower risk of risk of AD |
| Kulmala, J.(56) | 2014 | The CAIDE study | Mean 30 | 3,559 | Mean 50 | 57 | Question | Poor physical fitness | With higher dementia risk |
| Buchman, A. S.(57) | 2012 | The Rush Memory and Aging Project | Mean 4 | 716 | Mean 82 | 76 | Actigraphy | Total daily physical activity | With lower risk of AD |
| Sabia, S.(58) | 2017 | The Whitehall II study | 28 | 10,308 | 35-55 | 33 | Questionnaire | Physical activity | No association with dementia |
| Tolppanen, A.(59) | 2015 | The CAIDE study | 28 | 3,559 | Mean 50 | 57 | Question | Leisure-time physical activity | With lower risk of AD |
| Benedict, C.(60) | 2015 | The Uppsala Longitudinal Study of Adult Men | 40 | 2,322 | 50 | 0 | Questions | Sleep disturbance | With higher risk of AD |
| Lutsey, P. L.(61) | 2018 | The Atherosclerosis Risk in Communities Study | 15 | 1,667 | Mean 63 | 53 | Polysomnography | OSA | With higher risk of AD |
| Westwood, A. J.(62) | 2017 | The Framingham Heart Study | 10 | 2,457 | Mean 72 | 57 | Self-report | Prolonged sleep duration | With an increased risk of AD |
| Chen, J. C.(63) | 2016 | The Women’s Health Initiative Memory Study | 13 | 7,444 | ≥65 | 100 | Self-report | Sleep duration | V-shaped association with MCI/dementia risk |
| Jørgensen, J. T.(64) | 2020 | The Danish Nurse Cohort | ≥6 | 28,731 | ≥44 | 100 | Questions | Shift work | With increased risk of dementia |
| Deal, J. A.(65) | 2020 | The Atherosclerosis Risk in Communities study | ≥12 | 13,002 | 63 | 57 | Self-report | Cigarette smoking and time of quitting | With benefits to the cognition |
| Choi, D.(66) | 2018 | The Korean National Health Insurance System-National Health Screening Cohort | 8 | 46,140 | ≥60 | 0 | Questionnaire | Smoking cessation | With reduced risk of AD |
| Mons, U.(67) | 2013 | The prospective statewide cohort study in Germany | 5 | 1,697 | ≥65 | 59 | Standardized  questionnaires | Smoking | With increased risk of cognitive impairment |
| Langballe, E. M.(68) | 2015 | The Nord-Trøndelag Health study | >27 | 40,435 | ≥38 | 51 | Questionnaire | Frequent alcohol drinking | With increased risk of AD |
| Sabia, S.(69) | 2018 | The Whitehall II study | 28 | 10,308 | 35-55 | 33 | Questionnaire | Alcohol consumption >14 units/week | With greater risk of dementia |
| Zhang, R.Y.(70) | 2020 | The Health and Retirement Study | 9 | 19,887 | 62 | 60 | Questions | Alcohol drinking (10-14 drinks/week) | With benefits to the cognition |
| Handing, E. P.(71) | 2015 | The population-based Swedish Twin Registry | >43 | 12,326 | Mean 54 | 56 | The Swedish Twin Registry questionnaire | Alcohol consumption (>12 grams/day) | With greater risk of dementia |
| Topiwala, A.(72) | 2017 | The Whitehall II cohort | >30 | 550 | Mean 43 | 20 | The CAGE screening questionnaire | Moderate alcohol consumption | With adverse effects on cognition |
| Sugiyama, K.(73) | 2016 | The Ohsaki Cohort 2006 Study | 6 | 13,137 | ≥65 | 55 | FFQ | Coffee consumption | With a lower risk of dementia |
| Tomata, Y.(74) | 2016 | The Ohsaki Cohort 2006 Study | 6 | 13,645 | ≥65 | 56 | FFQ | Green tea consumption | With a lower risk of incident dementia |
| Noguchi-Shinohara, M.(75) | 2014 | The Nakajima Project | Mean 5 | 490 | >60 | 67 | A self-administered questionnaire | Green tea consumption | With a lower incidence of cognitive decline |

AD = Alzheimer’s disease; The CAIDE study = = The Cardiovascular Risk Factors, Aging and Dementia study; OSA = obstructive sleep apnea; MCI = mild cognitive impairment; FFQ = Food-frequency questionnaire; CAGE = cut-annoyed-guilty-eye.

**Supplemental Table 4. Prospective studies of diets in associations with cognitive decline and Alzheimer’s disease.**

| **Author** | **Publication year** | **Studies** | **Follow-up(years)** | **No. of participants** | **Age at baseline (years)** | **Female (%)** | **Risk factor measurements** | **Risk factor** | **Outcomes** |
| --- | --- | --- | --- | --- | --- | --- | --- | --- | --- |
| Mélissa, G.(76) | 2020 | The Three-City Study | 12 | 2,777 | Mean 76 | 61 | FFQ and the 24-hour dietary recall | Refined carbohydrates | With higher risk of risk of AD |
| Tangney, C. C.(77) | 2014 | The Memory and Aging Project | ≥4 | 826 | Mean 82 | 74 | FFQ | DASH- and Mediterranean-like dietary patterns | With a slower rate of cognitive decline |
| Hosking, D. E.(78) | 2019 | The Personality and Total Health Through Life study | 12 | 1,220 | Mean 63 | NA | CSIRO-FFQ | The MIND and Mediterranean diet | With reduced risk of cognitive impairment |
| Morris, M. C.(79) | 2015 | The Rush Memory and Aging Project | ≥5 | 960 | About 81 | 75 | FFQ | The MIND diet | With slower rates of cognitive decline |
| Morris, M. C.(80) | 2015 | The Rush Memory and Aging Project | Mean 5 | 923 | About 81 | 75 | FFQ | Three dietary patterns | With reduced risk of risk of AD |
| Ozawa, M.(81) | 2016 | The Whitehall II cohort study | ≥10 | 5,083 | About 55 | 29 | FFQ | An inflammatory dietary pattern | With accelerated cognitive decline |
| Samieri, C.(82) | 2020 | The Three-City Study | 12 | 627 | Mean 78 | 74 | FFQ | A novel diet pattern | With benefits on cognition |
| Littlejohns, T. J.(83) | 2014 | The Cardiovascular Health Study | Mean 6 | 1,658 | Mean 74 | 69 | Using liquid chromatography-tandem mass spectrometry | Vitamin D deficiency | With an increased risk of AD |
| Miller, J. W.(84) | 2015 | The University of California at Davis Alzheimer’s Disease Center longitudinal community diversity study | Mean 5 | 382 | Mean 76 | 62 | Competitive  immunoassay | Low Vitamin D status | With accelerated decline in cognitive function domain |
| Zhao, C.(85) | 2020 | The Washington Heights-Inwood Columbia Aging Project | Mean 6 | 1,759 | ≥65 | 69 | FFQ | Higher vitamin D intake | With decreased dementia risk |
| Olsson, E.(86) | 2017 | The Uppsala Longitudinal Study | 18 | 1,182 | Mean 71 | 0 | High-performance liquid chromatography-mass spectrometry, 7-day dietary records, a vitamin D-synthesis genetic risk score | Vitamin D | No association with AD |
| Agarwal, P.(87) | 2019 | The Rush Memory and Aging Project | Mean 7 | 925 | Mean 81 | 75 | FFQ | Vitamin C and total flavonoids | With reduced risk of AD |
| Noguchi-Shinohara, M.(88) | 2018 | The Nakajima study | About 7 | 349 | ≥65 | 66 | High-performance liquid chromatography and fluorescence | Vitamin C and vitamin E | With reduced risk of cognitive decline |
| Holland, T. M.(89) | 2020 | The Rush Memory and Aging Project | Mean 6 | 921 | Mean 81 | 75 | FFQ | Dietary intakes of flavonols | With reduced risk of AD |
| Honda, T.(90) | 2019 | The Hisayama study | 10 | 1,628 | ≥60 | NA | Using gas chromatography/mass spectrometry | Serum elaidic acid | With a greater risk of AD |

AD = Alzheimer’s disease; FFQ = Food frequency questionnaire; DASH = Dietary Approach to Stop Hypertension; MIND = Mediterranean-DASH diet Intervention for Neurodegenerative Delay; CSIRO-FFQ = Commonwealth Scientific and Industrial Research Organization semi-quantitative food frequency questionnaire

1. Gottesman RF, Albert MS, Alonso A, et al. Associations Between Midlife Vascular Risk Factors and 25-Year Incident Dementia in the Atherosclerosis Risk in Communities (ARIC) Cohort. JAMA neurology 2017;74: 1246-1254.

2. Wilson RS, Segawa E, Boyle PA, Bennett DA Influence of late-life cognitive activity on cognitive health. Neurology 2012;78: 1123-1129.

3. Najar J, Östling S, Gudmundsson P, et al. Cognitive and physical activity and dementia: A 44-year longitudinal population study of women. Neurology 2019;92: e1322-e1330.

4. Marioni RE, Proust-Lima C, Amieva H, et al. Social activity, cognitive decline and dementia risk: a 20-year prospective cohort study. BMC public health 2015;15: 1089.

5. Zhou Z, Wang P, Fang Y Social Engagement and Its Change are Associated with Dementia Risk among Chinese Older Adults: A Longitudinal Study. Scientific reports 2018;8: 1551.

6. Amieva H, Stoykova R, Matharan F, et al. What aspects of social network are protective for dementia? Not the quantity but the quality of social interactions is protective up to 15 years later. Psychosom Med 2010;72: 905-911.

7. Sommerlad A, Sabia S, Singh-Manoux A, Lewis G, Livingston G Association of social contact with dementia and cognition: 28-year follow-up of the Whitehall II cohort study. PLoS medicine 2019;16: e1002862.

8. Fancourt D, Steptoe A, Cadar D Community engagement and dementia risk: time-to-event analyses from a national cohort study. Journal of epidemiology and community health 2020;74: 71-77.

9. Biddle KD, Jacobs HIL, d'Oleire Uquillas F, et al. Associations of Widowhood and beta-Amyloid With Cognitive Decline in Cognitively Unimpaired Older Adults. JAMA network open 2020;3: e200121.

10. Saito T, Murata C, Saito M, Takeda T, Kondo K Influence of social relationship domains and their combinations on incident dementia: a prospective cohort study. Journal of epidemiology and community health 2018;72.

11. Geda YE, Roberts RO, Mielke MM, et al. Baseline neuropsychiatric symptoms and the risk of incident mild cognitive impairment: a population-based study. The American journal of psychiatry 2014;171: 572-581.

12. Holmquist S, Nordström A, Nordström P The association of depression with subsequent dementia diagnosis: A Swedish nationwide cohort study from 1964 to 2016. PLoS medicine 2020;17: e1003016.

13. Almeida OP, Hankey GJ, Yeap BB, Golledge J, Flicker L Depression as a risk factor for cognitive impairment in later life: the Health In Men cohort study. International journal of geriatric psychiatry 2016;31: 412-420.

14. Almeida OP, Hankey GJ, Yeap BB, Golledge J, Flicker L Depression as a modifiable factor to decrease the risk of dementia. Translational psychiatry 2017;7: e1117.

15. Steenland K, Karnes C, Seals R, et al. Late-life depression as a risk factor for mild cognitive impairment or Alzheimer's disease in 30 US Alzheimer's disease centers. Journal of Alzheimer's disease : JAD 2012;31: 265-275.

16. Song H, Sieurin J, Wirdefeldt K, et al. Association of Stress-Related Disorders With Subsequent Neurodegenerative Diseases. JAMA neurology 2020.

17. Sindi S, Hagman G, Hakansson K, et al. Midlife Work-Related Stress Increases Dementia Risk in Later Life: The CAIDE 30-Year Study. The journals of gerontology. Series B, Psychological sciences and social sciences 2017;72: 1044-1053.

18. Wang H-X, Wahlberg M, Karp A, Winblad B, Fratiglioni L Psychosocial stress at work is associated with increased dementia risk in late life. Alzheimer's & dementia : the journal of the Alzheimer's Association 2012;8: 114-120.

19. Gradus JL, Horváth-Puhó E, Lash TL, et al. Stress Disorders and Dementia in the Danish Population. American journal of epidemiology 2018;188: 493-499.

20. Flatt JD, Gilsanz P, Quesenberry CP, Jr., Albers KB, Whitmer RA Post-traumatic stress disorder and risk of dementia among members of a health care delivery system. Alzheimer's & dementia : the journal of the Alzheimer's Association 2018;14: 28-34.

21. Wang T-Y, Wei H-T, Liou Y-J, et al. Risk for developing dementia among patients with posttraumatic stress disorder: A nationwide longitudinal study. Journal of Affective Disorders 2016;205: 306-310.

22. Marseglia A, Fratiglioni L, Kalpouzos G, et al. Prediabetes and diabetes accelerate cognitive decline and predict microvascular lesions: A population-based cohort study. Alzheimer's & dementia : the journal of the Alzheimer's Association 2019;15: 25-33.

23. Zheng F, Yan L, Yang Z, Zhong B, Xie W HbA1c, diabetes and cognitive decline: the English Longitudinal Study of Ageing. Diabetologia 2018;61: 839-848.

24. Marseglia A, Dahl Aslan AK, Fratiglioni L, et al. Cognitive Trajectories of Older Adults With Prediabetes and Diabetes: A Population-Based Cohort Study. The journals of gerontology. Series A, Biological sciences and medical sciences 2018;73: 400-406.

25. McGrath ER, Beiser AS, DeCarli C, et al. Blood pressure from mid- to late life and risk of incident dementia. Neurology 2017;89: 2447-2454.

26. Rajan KB, Barnes LL, Wilson RS, et al. Blood pressure and risk of incident Alzheimer's disease dementia by antihypertensive medications and APOE ε4 allele. Annals of neurology 2018;83: 935-944.

27. Oishi E, Ohara T, Sakata S, et al. Day-to-Day Blood Pressure Variability and Risk of Dementia in a General Japanese Elderly Population: The Hisayama Study. Circulation 2017;136: 516-525.

28. Yoo JE, Shin DW, Han K, et al. Blood Pressure Variability and the Risk of Dementia: A Nationwide Cohort Study. Hypertension (Dallas, Tex. : 1979) 2020;75: 982-990.

29. Schilling S, Tzourio C, Soumare A, et al. Differential associations of plasma lipids with incident dementia and dementia subtypes in the 3C Study: A longitudinal, population-based prospective cohort study. PLoS medicine 2017;14: e1002265.

30. Marcum ZA, Walker R, Bobb JF, et al. Serum Cholesterol and Incident Alzheimer's Disease: Findings from the Adult Changes in Thought Study. Journal of the American Geriatrics Society 2018;66: 2344-2352.

31. Svensson T, Sawada N, Mimura M, et al. The association between midlife serum high-density lipoprotein and mild cognitive impairment and dementia after 19 years of follow-up. Translational psychiatry 2019;9: 26.

32. Tolppanen AM, Ngandu T, Kareholt I, et al. Midlife and late-life body mass index and late-life dementia: results from a prospective population-based cohort. Journal of Alzheimer's disease : JAD 2014;38: 201-209.

33. LeBlanc ES, Rizzo JH, Pedula KL, et al. Weight Trajectory over 20 Years and Likelihood of Mild Cognitive Impairment or Dementia Among Older Women. Journal of the American Geriatrics Society 2017;65: 511-519.

34. Alhurani RE, Vassilaki M, Aakre JA, et al. Decline in Weight and Incident Mild Cognitive Impairment: Mayo Clinic Study of Aging. JAMA neurology 2016;73: 439-446.

35. Rusanen M, Kivipelto M, Levälahti E, et al. Heart diseases and long-term risk of dementia and Alzheimer's disease: a population-based CAIDE study. Journal of Alzheimer's disease : JAD 2014;42: 183-191.

36. Dublin S, Anderson ML, Haneuse SJ, et al. Atrial fibrillation and risk of dementia: a prospective cohort study. Journal of the American Geriatrics Society 2011;59: 1369-1375.

37. Kim D, Yang P-S, Yu HT, et al. Risk of dementia in stroke-free patients diagnosed with atrial fibrillation: data from a population-based cohort. European heart journal 2019;40: 2313-2323.

38. Jefferson AL, Beiser AS, Himali JJ, et al. Low cardiac index is associated with incident dementia and Alzheimer disease: the Framingham Heart Study. Circulation 2015;131: 1333-1339.

39. Akoudad S, Wolters FJ, Viswanathan A, et al. Association of Cerebral Microbleeds With Cognitive Decline and Dementia. JAMA neurology 2016;73: 934-943.

40. Romero JR, Beiser A, Himali JJ, et al. Cerebral microbleeds and risk of incident dementia: the Framingham Heart Study. Neurobiology of aging 2017;54: 94-99.

41. Fann JR, Ribe AR, Pedersen HS, et al. Long-term risk of dementia among people with traumatic brain injury in Denmark: a population-based observational cohort study. The lancet. Psychiatry 2018;5: 424-431.

42. Yaffe K, Lwi SJ, Hoang TD, et al. Military-related risk factors in female veterans and risk of dementia. Neurology 2019;92: e205-e211.

43. Chen S, Honda T, Ohara T, et al. Serum homocysteine and risk of dementia in Japan. Journal of neurology, neurosurgery, and psychiatry 2020;91: 540-546.

44. Hooshmand B, Refsum H, Smith AD, et al. Association of Methionine to Homocysteine Status With Brain Magnetic Resonance Imaging Measures and Risk of Dementia. JAMA psychiatry 2019.

45. Hooshmand B, Solomon A, Kåreholt I, et al. Homocysteine and holotranscobalamin and the risk of Alzheimer disease: a longitudinal study. Neurology 2010;75: 1408-1414.

46. Osler M, Christensen GT, Mortensen EL, et al. Hearing loss, cognitive ability, and dementia in men age 19-78 years. European journal of epidemiology 2019;34: 125-130.

47. Liu CM, Lee CT Association of Hearing Loss With Dementia. JAMA network open 2019;2: e198112.

48. Saito S, Ohi T, Murakami T, et al. Association between tooth loss and cognitive impairment in community-dwelling older Japanese adults: a 4-year prospective cohort study from the Ohasama study. BMC oral health 2018;18: 142.

49. Okamoto N, Morikawa M, Tomioka K, et al. Association between tooth loss and the development of mild memory impairment in the elderly: the Fujiwara-kyo Study. Journal of Alzheimer's disease : JAD 2015;44: 777-786.

50. Takeuchi K, Ohara T, Furuta M, et al. Tooth Loss and Risk of Dementia in the Community: the Hisayama Study. Journal of the American Geriatrics Society 2017;65: e95-e100.

51. Oudin A, Forsberg B, Adolfsson AN, et al. Traffic-Related Air Pollution and Dementia Incidence in Northern Sweden: A Longitudinal Study. Environmental health perspectives 2016;124: 306-312.

52. Chen H, Kwong JC, Copes R, et al. Exposure to ambient air pollution and the incidence of dementia: A population-based cohort study. Environment international 2017;108: 271-277.

53. Grande G, Ljungman PLS, Eneroth K, Bellander T, Rizzuto D Association Between Cardiovascular Disease and Long-term Exposure to Air Pollution With the Risk of Dementia. JAMA neurology 2020.

54. Oudin A, Segersson D, Adolfsson R, Forsberg B Association between air pollution from residential wood burning and dementia incidence in a longitudinal study in Northern Sweden. PloS one 2018;13: e0198283.

55. Kishimoto H, Ohara T, Hata J, et al. The long-term association between physical activity and risk of dementia in the community: the Hisayama Study. European journal of epidemiology 2016;31: 267-274.

56. Kulmala J, Solomon A, Kareholt I, et al. Association between mid- to late life physical fitness and dementia: evidence from the CAIDE study. Journal of internal medicine 2014;276: 296-307.

57. Buchman AS, Boyle PA, Yu L, et al. Total daily physical activity and the risk of AD and cognitive decline in older adults. Neurology 2012;78: 1323-1329.

58. Sabia S, Dugravot A, Dartigues JF, et al. Physical activity, cognitive decline, and risk of dementia: 28 year follow-up of Whitehall II cohort study. BMJ (Clinical research ed.) 2017;357: j2709.

59. Tolppanen A-M, Solomon A, Kulmala J, et al. Leisure-time physical activity from mid- to late life, body mass index, and risk of dementia. Alzheimer's & dementia : the journal of the Alzheimer's Association 2015;11.

60. Benedict C, Byberg L, Cedernaes J, et al. Self-reported sleep disturbance is associated with Alzheimer's disease risk in men. Alzheimer's & dementia : the journal of the Alzheimer's Association 2015;11: 1090-1097.

61. Lutsey PL, Misialek JR, Mosley TH, et al. Sleep characteristics and risk of dementia and Alzheimer's disease: The Atherosclerosis Risk in Communities Study. Alzheimer's & dementia : the journal of the Alzheimer's Association 2018;14: 157-166.

62. Westwood AJ, Beiser A, Jain N, et al. Prolonged sleep duration as a marker of early neurodegeneration predicting incident dementia. Neurology 2017;88: 1172-1179.

63. Chen J-C, Espeland MA, Brunner RL, et al. Sleep duration, cognitive decline, and dementia risk in older women. Alzheimer's & dementia : the journal of the Alzheimer's Association 2016;12: 21-33.

64. Jørgensen JT, Hansen J, Westendorp RGJ, et al. Shift work and incidence of dementia: A Danish Nurse Cohort study. Alzheimer's & dementia : the journal of the Alzheimer's Association 2020.

65. Deal JA, Power MC, Palta P, et al. Relationship of Cigarette Smoking and Time of Quitting with Incident Dementia and Cognitive Decline. Journal of the American Geriatrics Society 2020;68: 337-345.

66. Choi D, Choi S, Park SM Effect of smoking cessation on the risk of dementia: a longitudinal study. Ann Clin Transl Neurol 2018;5: 1192-1199.

67. Mons U, Schöttker B, Müller H, Kliegel M, Brenner H History of lifetime smoking, smoking cessation and cognitive function in the elderly population. European journal of epidemiology 2013;28: 823-831.

68. Langballe EM, Ask H, Holmen J, et al. Alcohol consumption and risk of dementia up to 27 years later in a large, population-based sample: the HUNT study, Norway. European journal of epidemiology 2015;30: 1049-1056.

69. Sabia S, Fayosse A, Dumurgier J, et al. Alcohol consumption and risk of dementia: 23 year follow-up of Whitehall II cohort study. BMJ (Clinical research ed.) 2018;362: k2927.

70. Zhang R, Shen L, Miles T, et al. Association of Low to Moderate Alcohol Drinking With Cognitive Functions From Middle to Older Age Among US Adults. JAMA network open 2020;3: e207922.

71. Handing EP, Andel R, Kadlecova P, Gatz M, Pedersen NL Midlife Alcohol Consumption and Risk of Dementia Over 43 Years of Follow-Up: A Population-Based Study From the Swedish Twin Registry. The journals of gerontology. Series A, Biological sciences and medical sciences 2015;70: 1248-1254.

72. Topiwala A, Allan CL, Valkanova V, et al. Moderate alcohol consumption as risk factor for adverse brain outcomes and cognitive decline: longitudinal cohort study. BMJ (Clinical research ed.) 2017;357: j2353.

73. Sugiyama K, Tomata Y, Kaiho Y, et al. Association between Coffee Consumption and Incident Risk of Disabling Dementia in Elderly Japanese: The Ohsaki Cohort 2006 Study. Journal of Alzheimer's disease : JAD 2016;50: 491-500.

74. Tomata Y, Sugiyama K, Kaiho Y, et al. Green Tea Consumption and the Risk of Incident Dementia in Elderly Japanese: The Ohsaki Cohort 2006 Study. The American journal of geriatric psychiatry : official journal of the American Association for Geriatric Psychiatry 2016;24: 881-889.

75. Noguchi-Shinohara M, Yuki S, Dohmoto C, et al. Consumption of green tea, but not black tea or coffee, is associated with reduced risk of cognitive decline. PloS one 2014;9: e96013.

76. Gentreau M, Chuy V, Féart C, et al. Refined carbohydrate-rich diet is associated with long-term risk of dementia and Alzheimer's disease in apolipoprotein E ε4 allele carriers. Alzheimer's & dementia : the journal of the Alzheimer's Association 2020.

77. Tangney CC, Li H, Wang Y, et al. Relation of DASH- and Mediterranean-like dietary patterns to cognitive decline in older persons. Neurology 2014;83: 1410-1416.

78. Hosking DE, Eramudugolla R, Cherbuin N, Anstey KJ MIND not Mediterranean diet related to 12-year incidence of cognitive impairment in an Australian longitudinal cohort study. Alzheimer's & dementia : the journal of the Alzheimer's Association 2019;15: 581-589.

79. Morris MC, Tangney CC, Wang Y, et al. MIND diet slows cognitive decline with aging. Alzheimer's & dementia : the journal of the Alzheimer's Association 2015;11: 1015-1022.

80. Morris MC, Tangney CC, Wang Y, et al. MIND diet associated with reduced incidence of Alzheimer's disease. Alzheimer's & dementia : the journal of the Alzheimer's Association 2015;11: 1007-1014.

81. Ozawa M, Shipley M, Kivimaki M, Singh-Manoux A, Brunner EJ Dietary pattern, inflammation and cognitive decline: The Whitehall II prospective cohort study. Clinical nutrition (Edinburgh, Scotland) 2016;36: 506-512.

82. Samieri C, Rajendra Sonawane A, Lefèvre-Arbogast S, et al. Using network science tools to identify novel diet patterns in prodromal dementia. Neurology 2020: 10.1212/WNL.0000000000009399.

83. Littlejohns TJ, Henley WE, Lang IA, et al. Vitamin D and the risk of dementia and Alzheimer disease. Neurology 2014;83: 920-928.

84. Miller JW, Harvey DJ, Beckett LA, et al. Vitamin D Status and Rates of Cognitive Decline in a Multiethnic Cohort of Older Adults. JAMA neurology 2015;72: 1295-1303.

85. Zhao C, Tsapanou A, Manly J, et al. Vitamin D intake is associated with dementia risk in the Washington Heights-Inwood Columbia Aging Project (WHICAP). Alzheimer's & dementia : the journal of the Alzheimer's Association 2020.

86. Olsson E, Byberg L, Karlstrom B, et al. Vitamin D is not associated with incident dementia or cognitive impairment: an 18-y follow-up study in community-living old men. The American journal of clinical nutrition 2017;105: 936-943.

87. Agarwal P, Holland TM, Wang Y, Bennett DA, Morris MC Association of Strawberries and Anthocyanidin Intake with Alzheimer's Dementia Risk. Nutrients 2019;11.

88. Noguchi-Shinohara M, Abe C, Yuki-Nozaki S, et al. Higher Blood Vitamin C Levels are Associated with Reduction of Apolipoprotein E E4-related Risks of Cognitive Decline in Women: The Nakajima Study. Journal of Alzheimer's disease : JAD 2018;63: 1289-1297.

89. Holland TM, Agarwal P, Wang Y, et al. Dietary flavonols and risk of Alzheimer dementia. Neurology 2020.

90. Honda T, Ohara T, Shinohara M, et al. Serum elaidic acid concentration and risk of dementia: The Hisayama Study. Neurology 2019;93: e2053-e2064.
